# Comparative Dynamics of Delta and Omicron SARS-CoV-2 Variants across and between California and Mexico

**DOI:** 10.3390/v14071494

**Published:** 2022-07-08

**Authors:** Sanjay R. Mehta, Davey M. Smith, Celia Boukadida, Antoine Chaillon

**Affiliations:** 1 Department of Medicine, University of California, San Diego, CA 92093, USA; srmehta@health.ucsd.edu (S.R.M.); d13smith@ucsd.edu (D.M.S.); 2 Veterans Affairs Health System, San Diego, CA 92093, USA; 3 Centro de Investigación en Enfermedades Infecciosas, Instituto Nacional de Enfermedades Respiratorias Ismael Cosío Villegas, Ciudad de México 14080, Mexico; celia.boukadida@cieni.org.mx

**Keywords:** COVID-19, SARS-CoV-2, variants, Delta, Omicron, phylogeography

## Abstract

Evolutionary analysis using viral sequence data can elucidate the epidemiology of transmission. Using publicly available SARS-CoV-2 sequence and epidemiological data, we developed discrete phylogeographic models to interrogate the emergence and dispersal of the Delta and Omicron variants in 2021 between and across California and Mexico. External introductions of Delta and Omicron in the region peaked in early July (2021-07-10 [95% CI: 2021-04-20, 2021-11-01]) and mid-December (2021-12-15 [95% CI: 2021-11-14, 2022-01-09]), respectively, 3 months and 2 weeks after first detection. These repeated introductions coincided with domestic migration events with no evidence of a unique transmission hub. The spread of Omicron was most consistent with gravity centric patterns within Mexico. While cross-border events accounted for only 5.1% [95% CI: 4.3–6] of all Delta migration events, they accounted for 20.6% [95% CI: 12.4–29] of Omicron movements, paralleling the increase in international travel observed in late 2021. Our investigations of the Delta and Omicron epidemics in the California/Mexico region illustrate the complex interplay and the multiplicity of viral and structural factors that need to be considered to limit viral spread, even as vaccination is reducing disease burden. Understanding viral transmission patterns may help intra-governmental responses to viral epidemics.

## 1. Introduction

Identifying and tracking new SARS-CoV-2 variants is critical for public health efforts. Previous studies have shown that in the early phase of epidemic spread, intensive surveillance and interventions reduced transmission rates and even prevented the establishment of SARS-CoV-2 in some locations [1,2]. However, as the infectiousness of SARS-CoV-2 has increased with new variants, patterns of transmission have evolved, requiring a reconsideration of interventions. To help in this effort, scientists from around the world have curated and made publicly available over 8 million complete viral sequences since the start of the COVID-19 pandemic. As these genomic data are being collected and published in near real-time, this also enables the monitoring of the evolution of the virus across the world at an unprecedented speed and scale [3]. As a result, the Delta variant, first detected in India in October 2020, was classified as a variant of concern (VOC) by the World Health Organization (WHO) in May 2021 while the Omicron variant, first detected in South Africa and Botswana in November 2021, was designated as a VOC that same month. In addition, the Centers for Disease Control (CDC) used these genomic data to track the rapid rise of the Delta variant of concern (VOC) in June–July 2021 (>50% circulating SARS-CoV-2 in the USA at the end of June, and >95% at the end of July). These data were used to inform a coordinated approach to combat the pandemic, including temporary travel restriction and the extension of US–Mexico land border closure to non-essential travel [4] in July 2021 until 8 November 2021, when travel resumed for fully vaccinated foreign travelers to the USA. These data also identified the emergence of the Omicron VOC (first detected on 1 December in the USA) and its rapid rise in the following weeks (>50% of viruses in the USA for the week ending 25 December and >99% at the week ending 22 January 2022, while the relative proportion of Delta dropped to <1% of cases) [5].

Publicly available genomic data can also be used to estimate epidemiologic characteristics and the spatiotemporal dynamics of viral spread [6]. Furthermore, since the evolution of a single VOC is relatively slow and steady (~9 × 10^−4^ mutations per site per year [7]), Bayesian phylogeographic inferences from sequences that have been annotated with geographic origin and time can provide a framework to gain insight into the epidemiology of these SARS-CoV-2 variants [8,9,10]. The spatial heterogeneity in these transmission patterns can also impact the intensity of cases and population level immunity [7,11].

In this study, we use all publicly annotated full-length SARS-CoV-2 sequences available on GISAID (Global Initiative on Sharing All Influenza Data, GISAID public database [12]) as of 31 January 2022 to understand differences in the dispersal of Delta and Omicron across and between Mexico and CA, USA. We focus on these areas because Mexico and the US state of California are tightly linked culturally and commercially. For example, the San Diego–Tijuana land border alone has over 45 million crossings annually [13]. Additionally, as the most populated state in the USA with a robust economy, California also has cultural and economic ties to much of the rest of the world. For this reason, when new pandemics enter the USA (e.g., HIV, H1N1 influenza), California is usually one of the earliest USA states to be affected. Similarly, Mexico City, as an economic and cultural hub with ties across the world, is often impacted early in pandemics.

## 2. Materials and Methods

### 2.1. Compilation of Datasets and Data Preparation for Phylogenetic Inference

We analyzed full-length SARS-CoV-2 genomes with known dates of sampling collected on or before 31 January 2022 and made available on the GISAID public database [12]. From this dataset, we extracted Delta and Omicron variants based on the variant assignment provided on GISAID. Sequences with missing or incomplete dates of sampling or geospatial information were excluded. The remaining sequences were curated and aligned using Nextalign implemented in Nextclade [14]. To allow the simultaneous viewing of sampling dates and geographic information, data were uploaded to the MicroReact platform [15] under https://microreact.org/project/rqnwd9nymcvdkvq1dktbfj and https://microreact.org/project/d5mbblhkwgxctofu6wdtxt for Delta and Omicron, respectively.

### 2.2. Subsampling

The massive amount of available viral genomic data provides a unique opportunity to gain valuable real-time insight into dispersal dynamics, but it also leads to computational challenges and is not necessarily representative, as testing and sequencing efforts vary through time and between locations. Hence, in Supplementary Table S1 and Figures S1 and S2, we show a higher number of infections sequenced in San Diego and Los Angeles Counties compared to other counties in California and across Mexico, highlighting the lack of correlation between cases and sampling depth. These structural factors (e.g., testing efforts, sequencing capacity) may bias attempts to accurately model these epidemics. To tackle these limitations and optimize the phylogenetic inferences described below, we applied a subsampling strategy to enhance the computational feasibility of our phylogeographic analyses. This strategy was informed by the COVID-19 epidemic in the California–Mexico region. First, we gathered the number of confirmed COVID-19 cases in Californian counties [16] and Mexican states [17] since January 2021. Next, we normalized reported cases per geospatial unit to the population size (i.e., number of cases per 100,000 individuals, see Supplementary Figure S3). To obtain datasets that reflected the local dynamics of the past year and limit sampling bias (i.e., the over-representation of sequences from a particular location due to increased sequencing effort), we applied a random subsampling strategy informed by the number of cases/100k individuals per month (for Delta sequences) or week (for Omicron sequences).

### 2.3. Lineage Identification

**Figure 1:**
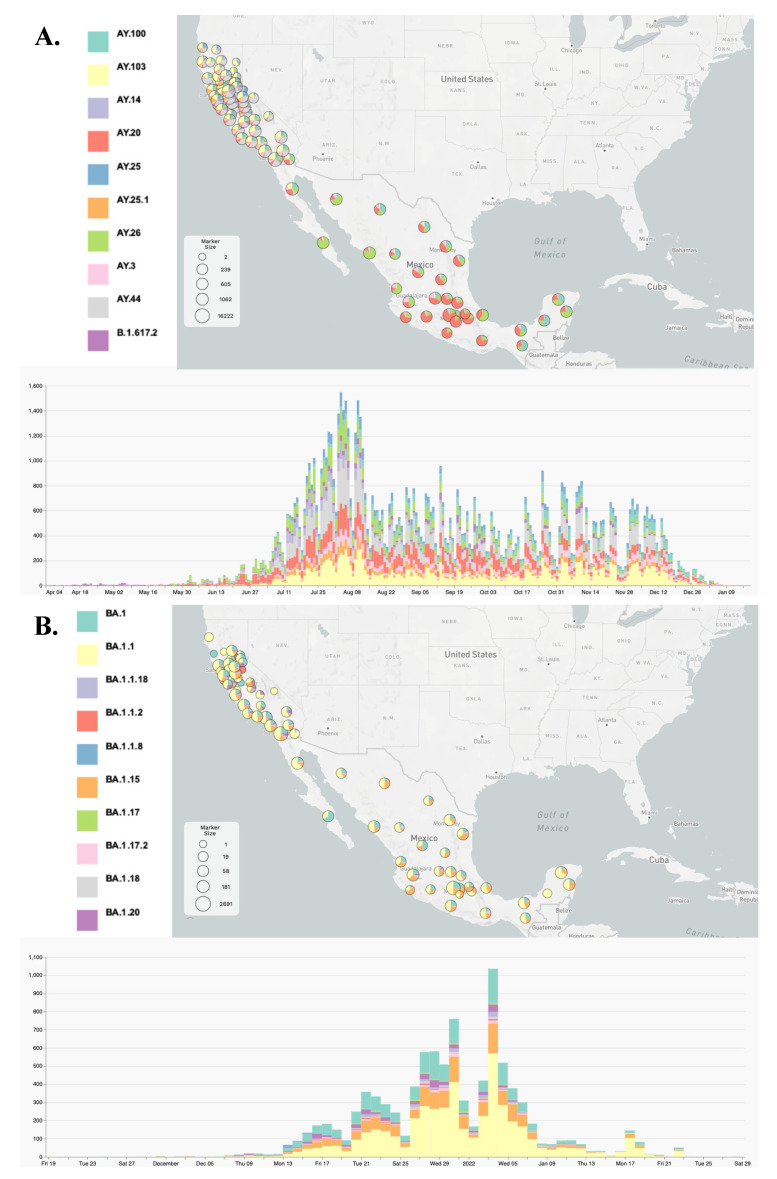
Visualization of the ten most prevalent Delta (**A**) and Omicron (**B**) genomes using MicroReact. MicroReact [15] display of the geolocation of the ten most prevalent genomes with associated lineage inferred by the Pangolin assignment tool [18]. See also Supplementary Figure S4 and MicroReact projects https://microreact.org/project/rqnwd9nymcvdkvq1dktbfj and https://microreact.org/project/d5mbblhkwgxctofu6wdtxt for all circulating Delta and Omicron sub-variants, respectively.

We used the Phylogenetic Assignment of Named Global Outbreak Lineages (Pangolin) (version 1.1.14) command-line tool and PANGO nomenclature system [18] to determine the lineage of all SARS-CoV2 genomes. The distribution lineages by Californian counties and Mexican states are provided in Figure 1 and Figure S4 using the MicroReact projects noted.

### 2.4. Identification of Representative Phylogenetic Clades

We applied the following step-by-step analytical approach [19]: using the final alignments for each subset of Delta and Omicron sequences combined with their respective background described above, a maximum likelihood phylogenetic tree was inferred using FastTree 2 [20] under a GTR+Γ evolutionary model. All clades within the trees were analyzed to delineate the multiplicity and timing of external introductions of COVID-19 in the region for each VOC. Of those, clades of size ≥ 5 including sequences originating from ≥2 distinct locations (i.e., Californian counties and Mexican states) were retained for further evaluating the dispersal of both epidemics within California and Mexico and across the California–Mexico border.

### 2.5. Timing of External Introductions

For each clade, the phylogeny was rescaled into units of time with treedater [21], assuming a strict molecular clock with the rate of SARS-CoV-2 genome evolution (of a single VOC) drawn from an externally estimated distribution [7]. Specifically, a normal distribution was specified with mean 9.41 × 10^−4^ nucleotide substitutions per site per year and a standard deviation of 4.99 × 10^−5^ [7]. This allowed us to infer the time to the most recent common ancestor (tMRCA) of each clade, which reflects the timing of that particular introduction. To incorporate uncertainty in the estimated clock rate, molecular clock estimation was replicated 100 times and the average timing of introduction for each clade was inferred.

### 2.6. Phylogeographic Inference

Phylogeographic inference was performed using the asymmetric discrete trait diffusion model and a GTR+Γ substitution model [22] implemented in the BEAST 1.10.5 software package [23]. Briefly, we employed a Metropolis–Hastings algorithm for MCMC chains that required 3 parallel runs for Omicron and 10 parallel runs for Delta variants that were run for sufficiently long (from 1.9 × 10^8^ to 3.5 × 10^9^ iterations) to obtain enough samples from the posterior distribution in order to reach adequate ESS values as estimated by the program Tracer v1.7 [24]. Trees were sampled every 250,000 iterations and 10% of the sampled trees were discarded as burn-in. Estimates of the posterior probability of the expected number of variant introduction and migration events between all pairs of locations (Markov jumps) were computed through stochastic mapping techniques [25]. To identify the subset of transition rates that was most informative to reconstruct the dispersal history and assess its significance, we applied a measure of statistical support associated with a lower false-positive rate by incorporating information on the relative abundance of samples from each geospatial location in the data set, which was coined as the adjusted Bayes factor (BF_adj_; see our publication [26] for the detailed procedure). In our analyses, we only considered variant migration events associated with a BF_adj_ support ≥ 3 as evidence of positive support [27].

### 2.7. Mobility Data

To estimate human mobility patterns across the California–Mexico border, we analyzed travel pattern edges between the United States and Mexico provided by Facebook [28]. These data can be used to provide insight into factors impacting viral migration, as shown in the UK [29]. These data are based on the number of Facebook users moving over large distances, such as air or train travel. Counts of international travel events are updated daily based only on users who have opted to share precise location data from their device with the Facebook mobile app through location services.

## 3. Results

### 3.1. Clade Identification and Variant External Introduction in the Border Region

We analyzed 110,378 and 9610 SARS-CoV-2 complete genome sequences identified as Delta and Omicron VOCs sampled in California (n = 86,688 and 5656, respectively) and Mexico (n = 23,690 and 3954, respectively) in combination with a set of representative background sequences sampled outside of the California-Mexico region(n = 88,826 and 19,531 Delta and Omicron complete genomes available). See Supplementary Figure S1 and Table S1 and MicroReact projects https://microreact.org/project/rqnwd9nymcvdkvq1dktbfj and https://microreact.org/project/d5mbblhkwgxctofu6wdtxt for Delta and Omicron, respectively. To accommodate the computational challenges with the large dataset, we generated ten random subsets of Delta and Omicron sequences from California/Mexico (Supplementary Table S2). These random subsets were combined with a background set of sequences from outside the region with a maxim 3.2 um of ten sequences per country per month for Delta and per week for Omicron, and were used to perform discrete phylogeographic analyses to identify the ancestral internal nodes of distinct descending clades of infection in the California–Mexico region.

**Figure 2:**
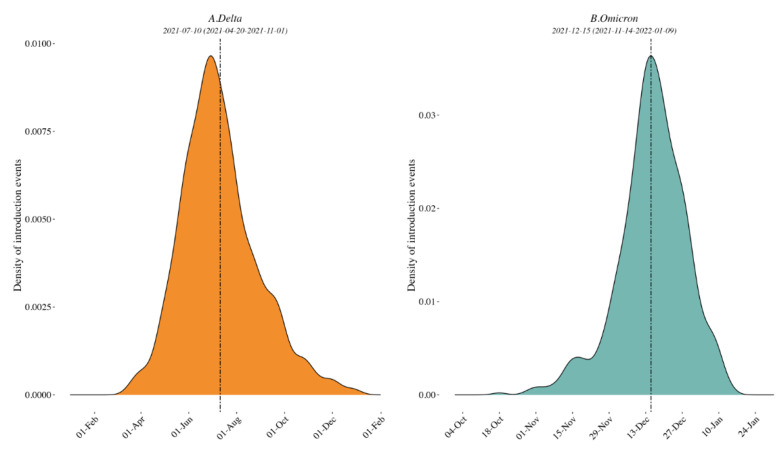
Density plots of external introduction of the identified clades for Delta (**A**) and Omicron (**B**) variants. A normal distribution was specified for the evolutionary rate with a mean of 9.41 × 10^−4^ nucleotide substitutions per site per year and a standard deviation of 4.99 × 10^−5^ [7]. All 10 replicates are combined to show the timing of the introductions of the clades in the region. See also Supplementary Figure S5 for the results of each of the 10 individual replicates.

*Delta variant*. A mean of 747 [range: 744–753] sequences (79 locations, i.e., Californian counties or Mexican states) were included in each subsampled phylogeny along with a representative background dataset of external sequences, and we identified a mean of 101 clades of size ≥ 2 across 69 locations. The peak time of external introductions of Delta into California/Mexico was estimated to be in early July 2021 (2021-07-10 [95% CI: 2021-04-20, 2021-11-01]) for all down-sampled subsets (i.e., tMRCA, see Figure 2 and Figure S5).

*Omicron variant*. A mean of 642 [range: 631–661] sequences (64 locations, i.e., Californian county or Mexican state) were included in each subsampled phylogeny, and we identified a mean of 70 clades of size ≥ 2 across 25 locations. The peak time of introduction in the California–Mexico border region was estimated to be in mid-December 2021 across all data replicates (2021-12-15 [95% CI: 2021-11-14, 2022-01-09], see Figure 2 and Figure S5). Characteristics of each replicate dataset for each VOC are summarized in Supplementary Table S2.

### 3.2. Lineage Distribution of the Delta and Omicron Variants

Next, we analyzed the geographical distribution of Delta and Omicron lineages. After they emerged, the parental B.1.617.2 lineage (Delta variant) and B.1.1.529 lineage (Omicron variant) diversified into 245 and 111 lineages designated as AY and BA in the Pango nomenclature system, respectively. In general, California and Mexico exhibited notable differences in lineage distribution patterns (Figure 1 and Figure S4 and related MicroReact projects (links above) for Delta and Omicron, respectively). For Delta, California was dominated by the AY.44 and AY.103 lineages, with relative frequencies of 22.7% and 20.1%, respectively, while the AY.20 (45.15%) and AY.26 (23.2%) lineages were most prevalent in Mexico. Interestingly, the epidemiology in the state of Baja California (Mexico) was more similar to southern California (US), with a higher prevalence of AY.103 and a lower relative frequency of AY.20 and AY.26 compared to other Mexican states. The distribution of Omicron lineages was more similar between California and Mexico, with a predominance of BA1, BA1.1, and BA1.15.

### 3.3. Viral Dynamics within California and Mexico

To interrogate viral migration patterns in the California–Mexico border region, we used a mean of 32 clades of size ≥ 5 including ≥ 2 locations within our study region for Delta, and 10 clades of size ≥ 5 including ≥ 2 locations for Omicron.

**Figure 3:**
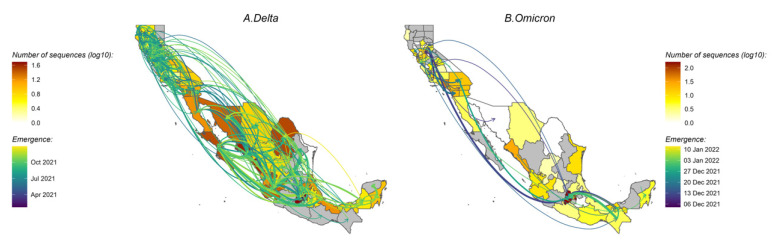
Map of migration events between locations for Delta (**A**) and Omicron (**B**) variants. The thickness of the arrows reflects the average number of inferred transmission events between locations and the colors of the arrows indicate the timing of the transmission event. California counties and Mexico states are colored according to the number of sequences. All 10 replicates are combined to show the timing of the introductions of the clades in the region. See also Supplementary Figure S6 for the results of each of the 10 replicates. Only the migration events with strong support (BF ≥ 20) are shown.

**Figure 4:**
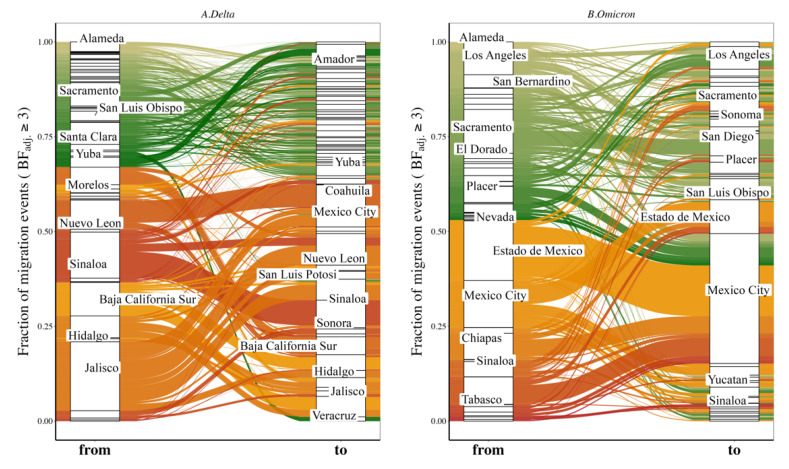
Proportion of viral migration events from each source location (‘from’) toward the recipient location (‘to’) for Delta (**A**) and Omicron (**B**) variants. The Sankey plot shows the proportion of viral migration events from each source location (Californian county or Mexican state) toward the recipient location. Left side of the plot shows the source location (‘from’) and right side of the plot shows the destination location (‘to’). We only report migration events associated with an adjusted Bayes factor (BFadj) ≥ 3. See also Supplementary Figure S7 for results of each of the 10 individual replicates. Locations accounting for ≥ 2% of all events are labeled.

**Figure 5:**
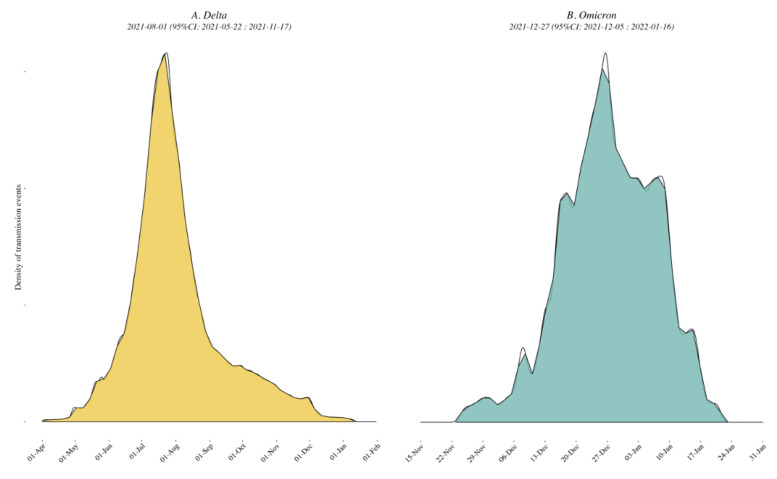
Probability density of variant migration events between locations for Delta (**A**) and Omicron (**B**) variants. Only migration events supported with an adjusted Bayes Factor ≥ 3 are considered. See also Supplementary Figure S8 for results of each of the 10 individual replicates.

*Delta variant*. Of the ten replicated discrete phylogeographic analyses, four showed the western state of Hidalgo and central states of Mexico City and Jalisco as a source of variant dispersal within Mexico. There was no clear predominant hub of dispersal observed in California (Figure 3A and Figure 4A; see also Supplementary Figures S6A and S7A for the results of each replicate). The variation across the ten replicates was likely due to the limited number of sequences included in each subset analysis, and is further discussed below. To analyze the viral diffusion processes more finely, we interrogated the density of variant migrations over time (Figure 5A and Figure S8A). Delta variant domestic migration events peaked in mid-summer 2021 and then progressively declined through the end of 2021 when Omicron was first identified. This coincided with the intensity of external introduction events.

*Omicron variant*. We again found that the city and state of Mexico (center south region) were important hubs of dispersal within Mexico (Figure 3B and Figure 4B; see also Supplementary Figures S6B and S7B). Similar to what we observed for Delta, the timing and intensity of Omicron local migrations and external variant introductions coincided and contributed simultaneously to the spatial dispersion of Omicron in the California–Mexico region (Figure 5B and Figure S8B) in December 2021.

### 3.4. Variant Migration across the California–Mexico Border

To evaluate the contribution of cross-border variant migration events (i.e., migration from California toward Mexico or from Mexico toward California) to the global dispersal of the Delta and Omicron epidemics, we evaluated the relative contribution of domestic and cross-border viral migrations.

**Figure 6:**
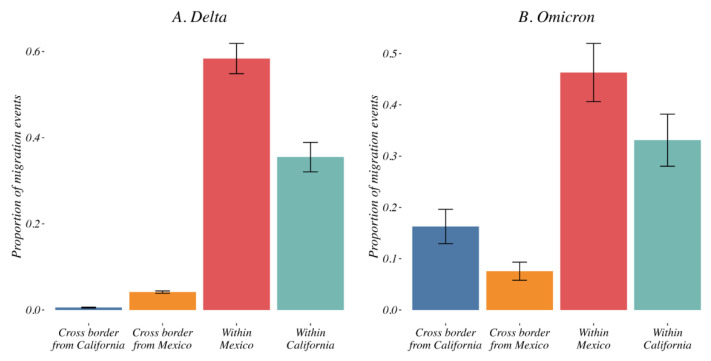
Relative contribution of national vs. international transmission events to the Delta (**A**) and Omicron (**B**) epidemics. The variant migration events across the border (from California toward Mexico and from Mexico toward California in blue and orange respectively) accounted for 3.4% and 23% of all inferred migration events for Delta and Omicron, respectively. Migration events within California and within Mexico are shown in red and green. See Supplementary Figure S9 for a description of these contributions over time.

*Delta variant*. During the Delta epidemic in 2021, the majority of viral movement was domestic, with a small amount of international migration in the border region with cross-border events accounting for 5.1% [95% CI: 4.31–6] of all inferred Delta variant migration events (mean of 0.92% [95% CI: 0.59–1] from California toward Mexico and 4.18% [95% CI: 3.58–5] from Mexico toward California, Figure 6A). Longitudinally, we showed that these cross-border transmissions were detected as early as May 2021 but remained limited with no peak thorough the study period (Supplementary Figure S9A).

*Omicron variant*. Distinct from what we observed for the Delta variant, we identified a high proportion of cross-border events for Omicron (from California toward Mexico and from Mexico toward California). We observed within the first weeks of December a sudden increase in cross-border viral migration accounting for 20.58% [95% CI: 12.38–29] of all inferred events (mean of 16.28% [95% CI: 8.37–24] from California toward Mexico and 7.56% [95% CI: 3.55–12] from Mexico toward California, Figure 6B). These cross-border migration events peaked in the last weeks of December 2021 (Supplementary Figure S9B).

### 3.5. Human Mobility and Variant Dispersal

To interrogate the role of human mobility, we examined the relationship between geospatial distance and migration events. As expected, we observed a negative correlation between distance and the intensity of transmission events (i.e., more events between close locations), and this association between proximity and dispersal was observed for both the Delta and Omicron variants (*p* = 0.01 and 0.06, respectively). Interestingly, while overall long-distance dispersal events were relatively infrequent (Supplementary Figure S10), they were more frequent for Omicron than for Delta, consistent with the inferences from our Bayesian models that showed a higher proportion of cross-border migration events for Omicron (Figure 6).

To assess the association between human mobility and cross-border variant movement, we analyzed Facebook international travel mobility data [28]. The density of variant migration events from California toward Mexico (green line) and from Mexico toward California (red line) increased as international travel increased in June–July 2021 and December 2021 (Supplementary Figure S11). While the increase in cross-border travel observed in June–July 2021 was not associated with significant cross-border variant movement for the Delta epidemic, the sharp increase in cross-border Omicron movement in late 2021 occurred when the number of international travel events peaked in December.

## 4. Discussion

Our phylogenetic investigations of the Delta and Omicron epidemics in the California/Mexico region illustrate a complex interplay between viral and structural forces for viral spread. The objective of our first phylogeographic analysis was to delineate clades corresponding to distinct viral introductions of SARS-CoV-2 Delta and Omicron lineages into the California/Mexico region. We found evidence of numerous external viral introductions of Delta and Omicron clades in the region throughout the study period. Of note, the probability density distribution of introduction events was highly negatively skewed from the peak for Omicron (−1.90) as compared to Delta (0.64; see Supplementary Table S3) with a high amount of kurtosis (13.8816) as compared to Delta (3.57), suggesting that the initial introduction of Omicron may have occurred much earlier than the estimated date of introduction, or similarly that lesser (limited propagation) introductions may have been occurring prior to the estimated peak introduction date. Discrete phylogeographic analyses of these clades demonstrated their contribution to the ongoing local circulation of Delta and Omicron in the region. Our results suggest that transmission chains circulating in California and Mexico were not established by a restricted number of isolated infectious cases but rather a combination of multiple external viral introductions, with cross-border and domestic variant movements occurring at the same time (Figure 2 and Figure 5). This observation is in line with other reports analyzing the first waves of the epidemic where no predominant lineage was identified [8,30]. However, this is distinct from what was seen by Kraemer et al. in the UK, where London acted as a dominant source of dispersal of B1.1.7 VOC [10], or in the USA, where New York was considered the location of emergence and expansion of VOC B.1.526 [8,31]. These differences likely reflect the fact that these two variants were first detected in London and New York and these cities acted as hubs. These results are consistent with a classic gravity model framework where large metropolitan areas act as the main transmission hub in an epidemic. Similarly, our analyses of the Omicron variant within Mexico showed that the city and state of Mexico (center south region) were important hubs of dispersal within Mexico. Our results were somewhat different for the Delta epidemic; while Mexico City and state were important, they were not singular predominant sources of viral dispersal. Rather, the epidemic was driven by a combination of repeated viral introductions from outside the region together with the circulation of multiple clades across Mexico.

We found that in the Mexican state of Baja California, the distribution of Delta lineages was more similar to southern California than central Mexico, highlighting the importance of proximity in the diffusion of these SARS-CoV-2 variants and suggesting cross-border transmission. Concerning Omicron, we noted a lower diversity and a more heterogenous distribution of lineages across California and Mexico, with BA.1.1, BA.1 and BA.1.15 being the most prevalent. This may reflect the more recent emergence and introduction of Omicron compared to Delta.

To untangle the factors leading to this complex epidemiology, we interrogated the contribution of domestic and international variant migration events. We found that cross-border viral movement contributed marginally to the Delta epidemic in mid-2021 (5.1% [95% CI: 4.31–6] of inferred migration events), while nearly a quarter of all inferred variant migration events identified for Omicron in late 2021 were cross-border. A small positive skewness was observed for Delta, possibly suggesting an ongoing trickle of migration during the Delta wave. The observed differences in the spatial invasion of the two major VOCs may be explained by a combination of viral and structural/behavioral factors. The shorter duration of the Omicron wave and time to peak migration may be a function of the increased transmissibility of the Omicron variant compared to the Delta variant, which may be a function of the increased affinity of the Omicron spike to the ACE2 receptor or overall hydrophobicity of the Omicron spike [32,33,34]. In addition to differences in variant characteristics (such as per-contact transmissibility, duration of infectiousness, and immune evasion [35,36]), local circulation and variant replacement dynamics are likely affected by human mobility but also by spatiotemporal heterogeneity in various structural factors (e.g., access and coverage of vaccinations) that need to be considered. Previous studies have shown that mobility data can be used to predict the spread of SARS-CoV2 [29,37,38] and other viruses [39]. In early phases of the COVID-19 pandemic, travel bans were effective in preventing COVID-19 importation [40,41]. Here, we interrogated the association between international viral migration and international travel using Facebook travel mobility data [28]. The increase in travel across the USA/Mexico border during the summer (Supplementary Figure S10) was not reflected in the cross-border migration of Delta. On the other hand, the emergence of the highly transmissible Omicron occurred in late 2021 when non-essential travel resumed for fully vaccinated foreign travelers to the US (effective as of 8 November 2021 [42], while no restrictions were in place for entry into Mexico). This timing also corresponded with the holiday travel season, and the Facebook data reflected these differences. In the case of Omicron, this increase in cross-border mobility was associated with significant cross-border viral migration.

Other structural factors, such as the heterogeneity of vaccination coverage between locations in 2021 [43] could have also impacted viral dynamics. As of 1 June 2021, when we observed a rise of Delta variant introductions and variant migrations between sampled locations, 55% of the population in California received at least one dose (46.9% fully vaccinated) while only 16.9% in Mexico (9.7% fully vaccinated) had received one dose [44]. At the time of the upsurge of Omicron cases in early December, vaccination coverage was 71.8% (64.4% fully vaccinated) in California and 59.1% (50% fully vaccinated) in Mexico. While this may appear counterintuitive, the intrinsic characteristics of Omicron [35,36] may be responsible for the rapid spread of Omicron in late 2021 despite higher rates of vaccination coverage.

An important limitation of this observational study is that these analyses are highly dependent on the data available. While the volume of genomic data available presents a unique opportunity to gain valuable real-time insight into viral dynamics, it also raises major computational challenges and potential biases. Failing to correct for ecologic and sampling bias can potentially lead to artifactual outcomes in phylogeographic reconstructions [45,46]. Here, we proposed a subsampling strategy informed by epidemiological data [8] to mitigate sampling bias. The lack of full congruence among the ten replicates that we observed was expected and is likely due to the relatively limited number of samples considered in each replicated analysis, as shown in other settings [8]. Random subsampling selection can also lead to different dispersal inferences, illustrating the impact of the sampled phylogenetic diversity in shaping the inferred dispersal history, as previously shown by Dellicour et al. in New York [8]. However, consistently across all replicates and for both Delta and Omicron, we showed a complex interplay of external introductions and domestic viral dispersal. With regard to our cross-border analyses, we relied on Facebook data which only note travel between countries and may not be totally representative of travel specifically between California and Mexico. Furthermore, these mobility data may not be representative, as Facebook use may vary geographically and demographically.

Our investigations of the Delta and Omicron epidemics in the California/Mexico region illustrate the complex interplay and the multiplicity of viral and structural factors that need to be considered to limit viral spread, even as vaccination is reducing disease burden. While our phylodynamic analysis provided insight into patterns of viral introductions into the region and migration across the border, future analysis using compartmental ordinary differential equation models could provide additional insight on how changes in transmission rates, vaccination rates and flow across the border could impact these and future SARS-CoV-2 variant epidemics in the border region [47,48,49,50,51,52,53,54,55]. Given the rapid observed diffusion of Delta, and then Omicron, as the infectiousness of successive variants increases, containment strategies would need to be increasingly extreme to reduce spread. This recent history suggests that containment approaches are unlikely to be successful for any further waves of the SARS-CoV-2 pandemic. Benefitting from our recent experience with SARS-CoV-2, we are undoubtedly better prepared to face the emergence of potential new viral pandemics, but untangling the driving forces of future epidemics and balancing the societal risks and benefits of containment vs. mitigation strategies will always remain a challenge.

## Data Availability

Data can be visualized with MicroReact projects https://microreact.org/project/rqnwd9nymcvdkvq1dktbfj and https://microreact.org/project/d5mbblhkwgxctofu6wdtxt.

## Supplementary Materials

The following supporting information can be downloaded at: https://www.mdpi.com/article/10.3390/v14071494/s1, Figure S1: Map of publicly available SARS-CoV-2 sequences; Figure S2: Number of COVID-19 confirmed cases and full-length genome sequences by location; Figure S3: Map of COVID-19 cases per 100,000 individuals up to 31 January 2022 [56]; Figure S4: Visualization of Delta (A) and Omicron (B) genomes using MicroReact; Figure S5: Density plots of external introduction of the identified clades for delta (Panel A) and omicron (Panel B) variants for the 10 replicates; Figure S6: Map of migration events between location for Delta (Panel A) and Omicron (Panel B) variants; Figure S7: Proportion of viral migration events from each source location (‘from’) toward the recipient location (‘to’) for Delta (Panel A) and Omicron (Panel B) variants; Figure S8: Probability Density of variant migration events between locations for Delta (Panel A) and Omicron (Panel B) variants; Figure S9: Relative contribution of national vs international transmission events to the Delta (Panel A) and Omicron (Panel B) epidemics overtime; Figure S10: Distribution of the geographic distances of phylogenetic lineage movement events for Delta (Panel A) and Omicron (Panel B) variants; Figure S11: Density of viral migration events across the USA Mexico border and international travels; Table S1: Distribution of full-length SARS-CoV2 genomes by county sampled in California and by state sampled in Mexico in 2021–2022. Table S2: Sampling overview; Table S3: Skewness and kurtosis of the distribution of the external introduction events and viral migrations for Omicron and Delta variants.
