# Supplementary material for: Comparative Dynamics of Delta and Omicron SARS-CoV-2 Variants across and between California and Mexico
Source: Viruses. 2022 Jul 8;14(7):1494. doi: 10.3390/v14071494 (PMC9317407; doi:10.3390/v14071494)

Supplementary Tables and Figures

Supplementary Table 1. Distribution of full-length SARS-CoV2 genomes by county sampled in California and by state sampled in Mexico in 2021-2022. Panel A. Delta variant, Panel B: Omicron variant.

Supplementary Table S2. Sampling overview of each replicate for Delta and Omicron.

Supplementary Table S3. Skewness and kurtosis of the distribution of the external introduction events and viral migrations for Omicron and Delta variants.

Supplementary Figure S1. Map of publicly available SARS-CoV-2 sequences that have been deposited in the GISAID database [12] as of January 31st, 2022, for the Delta variant (panel A) and the Omicron variant (panel B).

Supplementary Figure S2. Number of COVID-19 confirmed cases and full-length genome sequences by location. Scatter plot showing the number of confirmed cases per state per 100,000 individuals (X-axis) in California (left panel) and Mexico (right panel) vs. the number of cases sequenced (Y-axis) as of January 31st, 2022 (publicly available on GISAID). Locations with >10,000 sequences are labeled.

Supplementary Figure S3. Map of COVID-19 cases per 100,000 individuals up to January 31st 2022. Total number of confirmed COVID-19 cases per 100,000 individuals in Californian counties [16] and in Mexican states [17,56].

Supplementary Figure S4. Visualization of Delta (A) and Omicron (B) genomes using MicroReact. MicroReact [15] display of the geolocation of all genomes with associated lineage inferred by the Pangolin assignment tool [186]. See also **Figure 1** and MicroReact projects

https://microreact.org/project/rqnwd9nymcvdkvq1dktbfj and

https://microreact.org/project/d5mbblhkwgxctofu6wdtxt for Delta and Omicron respectively.

Supplementary Figure S5. Density plots of external introduction of the identified clades for delta (Panel A) and Omicron (Panel B) variants for the 10 replicates. A normal distribution was specified for the evolutionary rate with a mean 9.41×10^{-4} nucleotide substitutions per site per year and a standard deviation of 4.99×10^{-5} [7]. See also **Figure 2**.

Supplementary Figure S6. Map of migration events between location for Delta (Panel A) and Omicron (Panel B) variants. The thickness of the arrows reflects the average number of inferred transmission events between locations and the colors of the arrows indicate the timing of the transmission event. Locations are colored according to the number of sequences. See also **Figure 3.**

Supplementary Figure S7. Proportion of viral migration events from each source location ('from') toward the recipient location ('to') for Delta (Panel A) and Omicron (Panel B) variants. The Sankey plot shows the proportion of viral migration events from each source location (California county or Mexico state) toward the recipient location. Left side of the plot shows the source location (‘from') and right side of the plot shows the destination location ('to'). We only report migration events associated with an adjusted Bayes factor (BF_{adj}) ≥ 3.

Supplementary Figure S8. Probability Density of variant migration events between locations for Delta (Panel A) and Omicron (Panel B) variants. Only migration events supported with an adjusted Bayes Factor ≥3 are considered. See also **Figure 5**.

Supplementary Figure S9. Relative contribution of national vs. international transmission events to the Delta (Panel A) and Omicron (Panel B) epidemics overtime. See also **Figure 6** for the overall proportion of cross-border migration events.

Supplementary Figure S10. Distribution of the geographic distances of phylogenetic lineage movement events for Delta (Panel A) and Omicron (Panel B) variants. The panel on the left combines the results in all 10 replicates shown on the right.

Supplementary Figure S11. Density of viral migration events across the US–Mexico border and international travels. Probability density of international viral migration events between California and Mexico are represented in green and red. Blue and yellow lines represent the estimated counts of international travel (secondary y-axis) from the United States toward Mexico (blue) and Mexico toward the US (yellow) over time from the Facebook mobility data. Travel pattern edges between the United States and Mexico were provided by Facebook [28].

Supplementary Material. Phylogenies for each iteration and clade are available upon request to the authors.

Supplementary Table S1. Distribution of full-length SARS-CoV2 genomes by county sampled in California and by state sampled in Mexico in 2021-2022. Panel A: Delta variant; Panel B: Omicron variant.

A. Delta

Region	State or County	Number of sequences	Percentage	First sampling date	Last sampling date	Sampling duration
Mexico	Guanajuato	1,138	1.031	2021-06-09	2022-01-04	209 days
	Queretaro	543	0.492	2021-06-16	2022-01-05	203 days
	San Luis Potosi	345	0.313	2021-06-20	2022-01-03	197 days
	Zacatecas	386	0.350	2021-06-14	2022-01-11	211 days
	Estado de Mexico	2,149	1.947	2021-05-20	2022-01-05	230 days
	Mexico City	5,209	4.719	2021-01-27	2022-01-17	355 days
	Morelos	386	0.350	2021-01-21	2021-12-23	336 days
	Morelos	386	0.350	2021-01-21	2021-12-23	336 days
	Hidalgo	522	0.473	2021-06-10	2022-01-11	215 days
	Puebla	419	0.380	2021-05-23	2021-12-26	217 days
	Tlaxcala	95	0.086	2021-06-20	2021-12-30	193 days
	Veracruz	790	0.716	2021-06-08	2021-12-27	202 days
	Coahuila	599	0.543	2021-06-30	2022-01-03	187 days
	Nuevo Leon	802	0.727	2021-05-22	2021-12-31	223 days
	Tamaulipas	530	0.480	2021-07-02	2022-01-06	188 days
	Baja California	1,378	1.248	2021-06-12	2022-01-04	206 days
	Baja California Sur	433	0.392	2021-05-15	2022-01-04	234 days
	Chihuahua	625	0.566	2021-06-09	2022-01-03	208 days
	Durango	233	0.211	2021-06-24	2022-01-06	196 days
	Sinaloa	1,136	1.029	2021-05-06	2022-01-03	242 days
	Sonora	733	0.664	2021-06-15	2021-12-31	199 days
	Campeche	373	0.338	2021-06-14	2021-12-18	187 days
	Quintanaroo	650	0.589	2021-05-17	2022-01-02	230 days
	Tabasco	871	0.789	2021-06-17	2021-12-17	183 days
	Yucatan	928	0.841	2021-06-08	2022-01-09	215 days
	Chiapas	352	0.319	2021-06-30	2021-12-27	180 days
	Guerrero	147	0.133	2021-05-16	2021-12-24	222 days
	Oaxaca	241	0.218	2021-06-21	2022-01-06	199 days
	Colima	265	0.240	2021-06-25	2022-01-02	191 days
	Jalisco	788	0.714	2021-04-21	2022-01-04	258 days
	Michoacan	440	0.399	2021-05-11	2021-12-30	233 days
	Nayarit	184	0.167	2021-06-28	2022-01-03	189 days
California	Alameda	4,108	3.722	2021-01-24	2021-12-19	329 days
	Alpine	2	0.002	2021-09-07	2021-09-14	7 days
	Amador	295	0.267	2021-06-07	2021-12-11	187 days
	Butte	547	0.496	2021-06-14	2022-01-03	203 days
	Calaveras	208	0.188	2021-07-03	2021-12-18	168 days
	Colusa	9	0.008	2021-08-05	2021-12-01	118 days
	Contra Costa	2,371	2.148	2021-04-13	2021-12-27	258 days
	Del Norte	58	0.053	2021-07-22	2021-12-06	137 days
	El Dorado	512	0.464	2021-04-29	2021-12-17	232 days
	Fresno	2,871	2.601	2021-04-20	2021-12-30	254 days
	Glenn	137	0.124	2021-07-27	2021-12-04	130 days
	Humboldt	816	0.739	2021-06-03	2021-12-30	210 days
	Imperial	375	0.340	2021-06-14	2021-12-31	200 days
	Inyo	59	0.053	2021-07-23	2021-12-07	137 days
	Kern	1,135	1.028	2021-07-11	2021-12-26	168 days
	Kings	859	0.778	2021-06-07	2021-12-14	190 days
	Lake	409	0.371	2021-05-26	2021-12-30	218 days
	Lassen	6	0.005	2021-08-03	2021-08-25	22 days
	Los Angeles	8,066	7.308	2021-01-06	2022-01-03	362 days
	Madera	720	0.652	2021-05-04	2021-12-30	240 days
	Marin	338	0.306	2021-06-10	2021-12-18	191 days
	Mariposa	134	0.121	2021-07-22	2021-12-04	135 days
	Mendocino	739	0.670	2021-04-20	2021-12-22	246 days
	Merced	817	0.740	2021-04-27	2021-12-23	240 days
	Mono	128	0.116	2021-07-27	2021-12-09	135 days
	Monterey	1,098	0.995	2021-06-12	2022-01-09	211 days
	Napa	299	0.271	2021-06-10	2021-12-17	190 days
	Nevada	849	0.769	2021-05-07	2021-12-23	230 days
	Nevada	849	0.769	2021-05-07	2021-12-23	230 days
	Orange	2,677	2.425	2021-05-02	2022-01-07	250 days
	Placer	1,188	1.076	2021-05-02	2022-01-03	246 days
	Plumas	4	0.004	2021-06-02	2021-10-18	138 days
	Riverside	1,573	1.425	2021-06-03	2022-01-04	215 days
	Sacramento	5,482	4.967	2021-04-05	2022-01-06	276 days
	San Benito	236	0.214	2021-07-05	2022-01-03	182 days
	San Bernardino	1,519	1.376	2021-04-12	2021-12-27	259 days
	San Diego	20,250	18.346	2021-02-09	2022-01-14	339 days
	San Francisco	2,557	2.317	2021-04-15	2022-01-03	263 days
	San Joaquin	2,399	2.173	2021-05-04	2022-01-08	249 days
	San Luis Obispo	1,952	1.768	2021-04-26	2022-01-17	266 days
	San Mateo	722	0.654	2021-05-01	2021-12-18	231 days
	Santa Barbara	1,259	1.141	2021-06-16	2022-01-06	204 days
	Santa Clara	6,344	5.748	2021-04-07	2021-12-30	267 days
	Santa Cruz	917	0.831	2021-06-14	2022-01-06	206 days
	Shasta	242	0.219	2021-06-08	2021-12-03	178 days
	Sierra	7	0.006	2021-08-10	2021-12-01	113 days
	Siskiyou	36	0.033	2021-05-21	2021-12-15	208 days
	Solano	1,243	1.126	2021-04-19	2021-12-22	247 days
	Sonoma	1,849	1.675	2021-04-27	2022-01-10	258 days
	Stanislaus	1,755	1.590	2021-04-05	2021-12-20	259 days
	Sutter	260	0.236	2021-05-01	2021-12-27	240 days
	Tehama	199	0.180	2021-05-24	2021-12-03	193 days
	Trinity	36	0.033	2021-07-29	2021-12-02	126 days
	Tulare	751	0.680	2021-05-29	2021-12-30	215 days
	Tuolumne	524	0.475	2021-06-24	2021-12-18	177 days
	Ventura	1,208	1.094	2021-05-05	2021-12-29	238 days
	Yolo	1,272	1.152	2021-04-05	2021-12-27	266 days
	Yuba	262	0.237	2021-05-06	2021-12-18	226 days

B. Omicron

Region	State or County	Number of sequences	Percentage	First sampling date	Last sampling date	Sampling duration
Mexico	Guanajuato	24	0.249740	2021-12-27	2022-01-05	9 days
	Queretaro	50	0.520291	2021-12-15	2022-01-06	22 days
	San Luis Potosi	19	0.197711	2021-12-07	2022-01-06	30 days
	Zacatecas	49	0.509886	2021-12-14	2022-01-07	24 days
	Estado de Mexico	278	2.892820	2021-12-02	2022-01-22	51 days
	Mexico City	1,563	16.264308	2021-11-16	2022-01-28	73 days
	Morelos	2	0.020812	2021-12-28	2022-01-08	11 days
	Morelos	2	0.020812	2021-12-28	2022-01-08	11 days
	Hidalgo	26	0.270552	2021-12-19	2022-01-11	23 days
	Puebla	18	0.187305	2021-12-09	2022-01-09	31 days
	Tlaxcala	14	0.145682	2021-12-28	2022-01-06	9 days
	Veracruz	51	0.530697	2021-12-18	2022-01-10	23 days
	Coahuila	18	0.187305	2021-12-22	2022-01-06	15 days
	Nuevo Leon	110	1.144641	2021-12-18	2022-01-08	21 days
	Tamaulipas	150	1.560874	2021-12-03	2022-01-10	38 days
	Baja California	161	1.675338	2021-12-13	2022-01-06	24 days
	Baja California Sur	49	0.509886	2021-12-14	2022-01-05	22 days
	Chihuahua	64	0.665973	2021-12-24	2022-01-03	10 days
	Durango	17	0.176899	2021-12-24	2022-01-07	14 days
	Sinaloa	191	1.987513	2021-12-12	2022-01-07	26 days
	Sonora	39	0.405827	2021-12-21	2021-12-30	9 days
	Campeche	8	0.083247	2022-01-01	2022-01-02	1 days
	Quintanaroo	152	1.581686	2021-12-15	2022-01-10	26 days
	Tabasco	121	1.259105	2021-12-15	2022-01-10	26 days
	Yucatan	234	2.434964	2021-12-14	2022-01-10	27 days
	Chiapas	39	0.405827	2021-12-15	2022-01-10	26 days
	Guerrero	101	1.050989	2021-12-21	2022-01-10	20 days
	Oaxaca	65	0.676379	2021-12-21	2022-01-10	20 days
	Colima	10	0.104058	2021-12-22	2022-01-03	12 days
	Jalisco	269	2.799168	2021-12-13	2022-01-06	24 days
	Michoacan	13	0.135276	2021-12-24	2021-12-31	7 days
	Nayarit	49	0.509886	2021-12-28	2022-01-05	8 days
California	Alameda	37	0.385016	2021-11-30	2022-01-12	43 days
	Butte	72	0.749220	2021-12-17	2022-01-14	28 days
	Calaveras	1	0.010406	2021-12-28	2021-12-28	0 days
	Contra Costa	30	0.312175	2021-12-15	2022-01-22	38 days
	El Dorado	8	0.083247	2021-12-08	2022-01-09	32 days
	Fresno	7	0.072841	2021-12-09	2022-01-03	25 days
	Glenn	1	0.010406	2022-01-02	2022-01-02	0 days
	Humboldt	7	0.072841	2021-12-30	2022-01-02	3 days
	Imperial	20	0.208117	2021-12-13	2022-01-20	38 days
	Inyo	1	0.010406	2021-12-25	2021-12-25	0 days
	Kern	9	0.093652	2021-12-14	2022-01-03	20 days
	Lake	1	0.010406	2021-12-27	2021-12-27	0 days
	Los Angeles	554	5.764828	2021-11-27	2022-01-23	57 days
	Madera	7	0.072841	2021-12-29	2022-01-04	6 days
	Marin	7	0.072841	2021-12-16	2022-01-19	34 days
	Mendocino	1	0.010406	2021-12-14	2021-12-14	0 days
	Merced	1	0.010406	2021-12-28	2021-12-28	0 days
	Monterey	182	1.893861	2021-12-14	2022-01-09	26 days
	Napa	1	0.010406	2021-12-27	2021-12-27	0 days
	Nevada	16	0.166493	2021-12-08	2022-01-22	45 days
	Nevada	16	0.166493	2021-12-08	2022-01-22	45 days
	Orange	208	2.164412	2021-12-07	2022-01-22	46 days
	Placer	24	0.249740	2021-12-21	2022-01-14	24 days
	Riverside	58	0.603538	2021-12-13	2022-01-12	30 days
	Sacramento	92	0.957336	2021-12-02	2022-01-16	45 days
	San Benito	1	0.010406	2021-12-27	2021-12-27	0 days
	San Bernardino	47	0.489074	2021-12-08	2022-01-16	39 days
	San Diego	2,779	28.917794	2021-12-07	2022-01-20	44 days
	San Francisco	192	1.997919	2021-11-28	2022-01-04	37 days
	San Joaquin	203	2.112383	2021-12-19	2022-01-11	23 days
	San Luis Obispo	215	2.237253	2021-11-30	2022-01-20	51 days
	San Mateo	20	0.208117	2021-12-07	2022-01-23	47 days
	Santa Barbara	43	0.447451	2021-12-16	2022-01-04	19 days
	Santa Clara	340	3.537981	2021-12-02	2022-01-13	42 days
	Santa Cruz	17	0.176899	2021-12-16	2022-01-10	25 days
	Solano	4	0.041623	2021-12-17	2022-01-09	23 days
	Sonoma	243	2.528616	2021-12-13	2022-01-16	34 days
	Stanislaus	6	0.062435	2021-12-14	2022-01-10	27 days
		1	0.010406	2021-12-28	2021-12-28	0 days
	Sutter	5	0.052029	2021-12-14	2022-01-12	29 days
	Tulare	4	0.041623	2021-12-26	2021-12-31	5 days
	Ventura	102	1.061394	2021-12-12	2022-01-19	38 days
	Yolo	84	0.874089	2021-12-11	2022-01-09	29 days
	Yuba	2	0.020812	2022-01-06	2022-01-10	4 days

Supplementary Table S2. Sampling overview of each replicate for Delta and Omicron.

Variant	Replicates	Number of sequences	Number of locations	Number of sequences in clades	Number of clades	Number of locations in clades	Number of clades above 5	Number of dyads
Delta	Replicate 1	746	79	559	102	70	33	37
	Replicate 2	750	76	544	98	70	34	44
	Replicate 3	747	79	537	90	68	34	26
	Replicate 4	744	81	526	94	71	33	43
	Replicate 5	751	78	550	101	70	31	39
	Replicate 6	745	78	549	102	70	32	48
	Replicate 7	753	79	555	109	65	30	51
	Replicate 8	746	80	545	113	68	34	46
	Replicate 9	744	79	563	102	68	27	40
	Replicate 10	745	79	544	99	69	35	39
Omicron	Replicate 1	631	63	218	66	23	9	42
	Replicate 2	643	66	207	55	32	10	31
	Replicate 3	633	64	223	70	27	11	34
	Replicate 4	635	64	238	74	19	10	47
	Replicate 5	641	60	219	70	26	12	48
	Replicate 6	647	64	198	57	24	12	29
	Replicate 7	661	64	231	70	31	10	43
	Replicate 8	641	62	233	79	16	8	47
	Replicate 9	648	65	247	82	28	9	51
	Replicate 10	640	65	211	72	20	7	45

Supplementary Table S3. Skewness and kurtosis of the distribution of the external introduction events and viral migrations for Omicron and Delta variants.

	Variant	Skewness	Kurtosis
**Introductions**	Omicron	-1.90415	13.8816
	Delta	0.643226	3.57319
**Migration events**	Omicron	-0.289548	2.94777
	Delta	0.888538	4.08022

Supplementary Figure S1. Map of publicly available SARS-CoV-2 sequences from California and Mexico that have been deposited in the GISAID database [12] as of January 31st, 2022, for Delta variant (panel A) and Omicron variant (panel B).

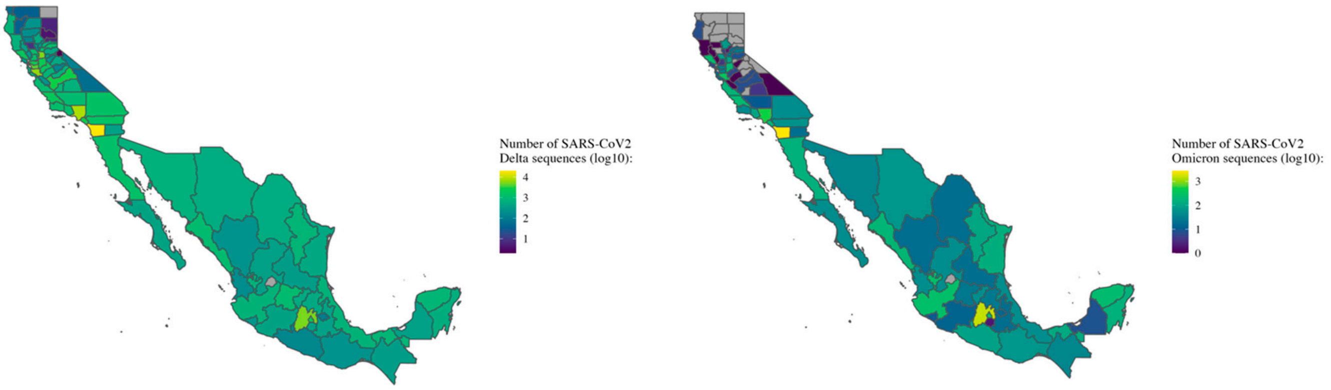

Supplementary Figure S2. Number of COVID-19 confirmed cases in relation to full-length SARS-CoV2 genome sequences by location.

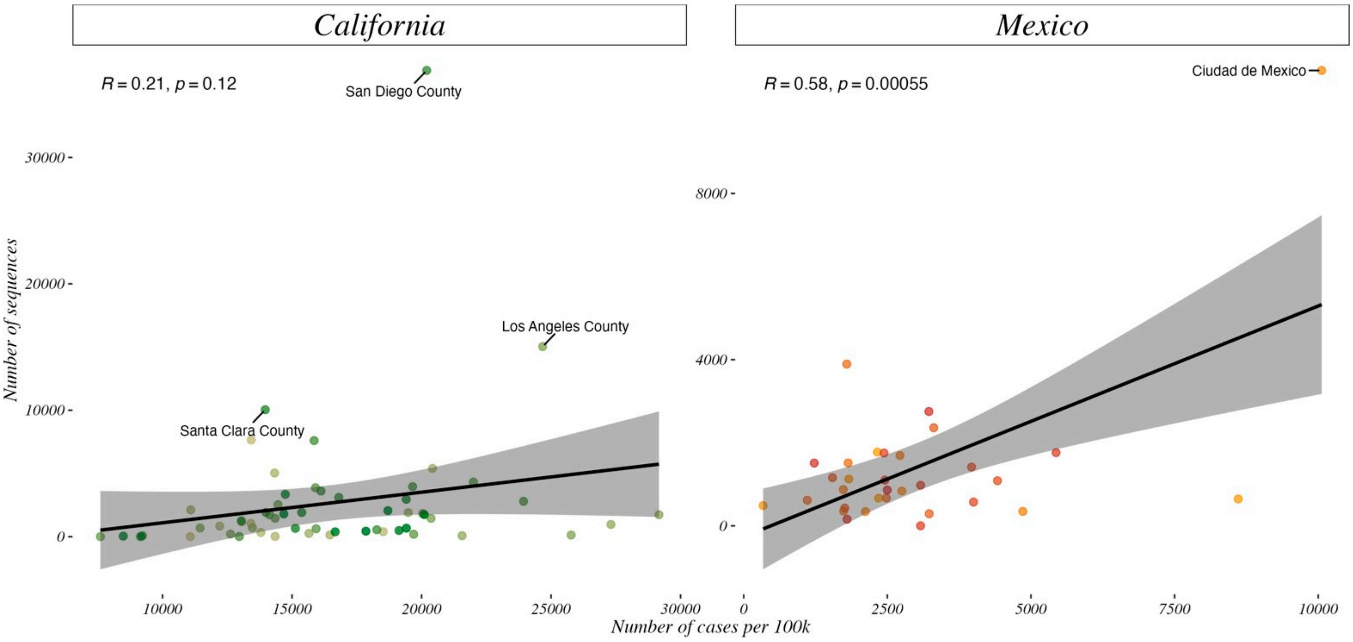

Supplementary figure S3. **Map of COVID-19 cases per 100,000 individuals up to January 31st 2022.**

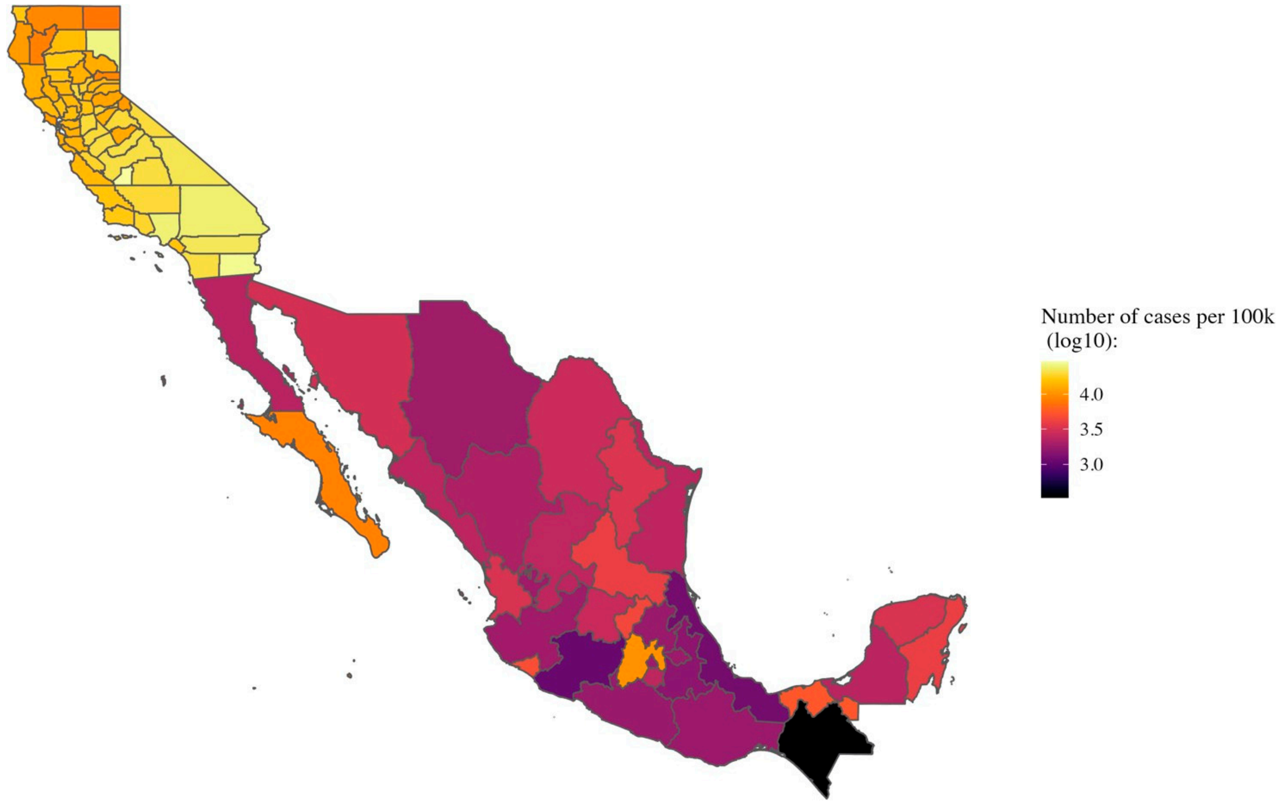

Supplementary figure S4. Visualization of Delta (A) and Omicron (B) genomes using MicroReact.

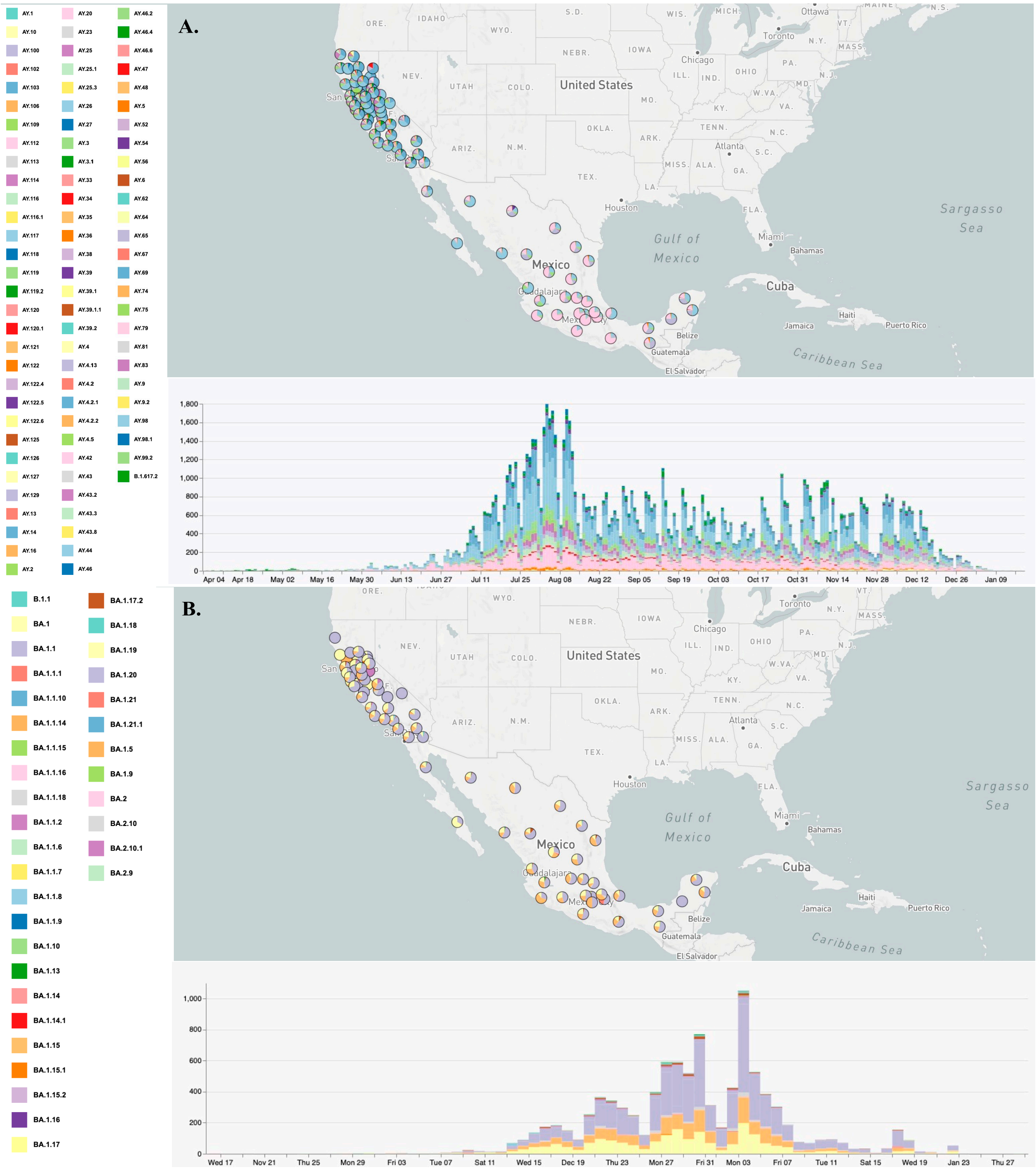

Supplementary Figure S5. Density plots of external introductions of the identified clades for delta (Panel A) and Omicron (Panel B) variants for the 10 replicates.

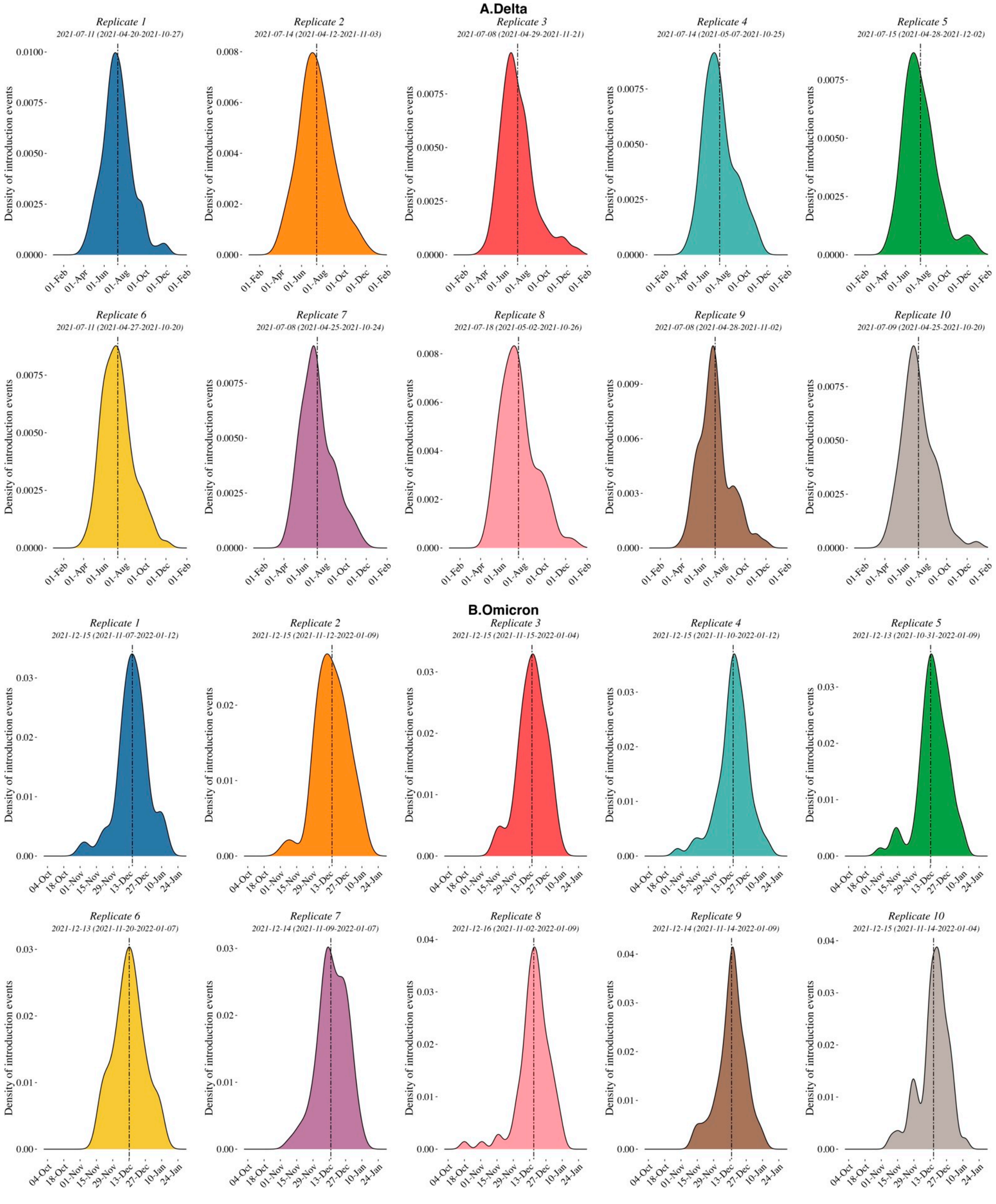

Supplementary Figure S6. Map of migration events between location for Delta (Panel A) and Omicron (Panel B) variants.

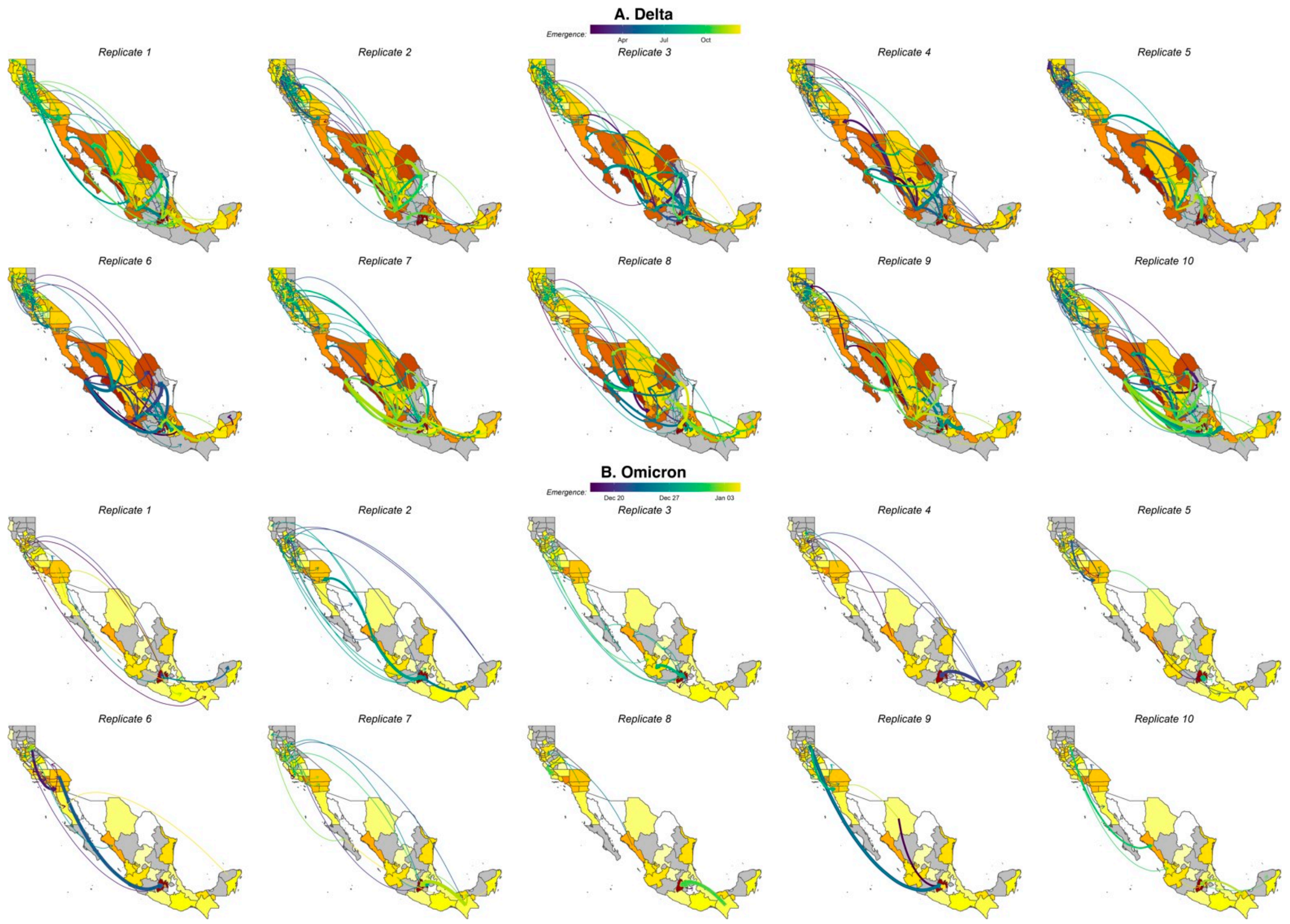

Supplementary Figure S7. Proportion of viral migration events from each source location ('from') toward the recipient location ('to') for Delta (Panel A) and Omicron (Panel B) variants.

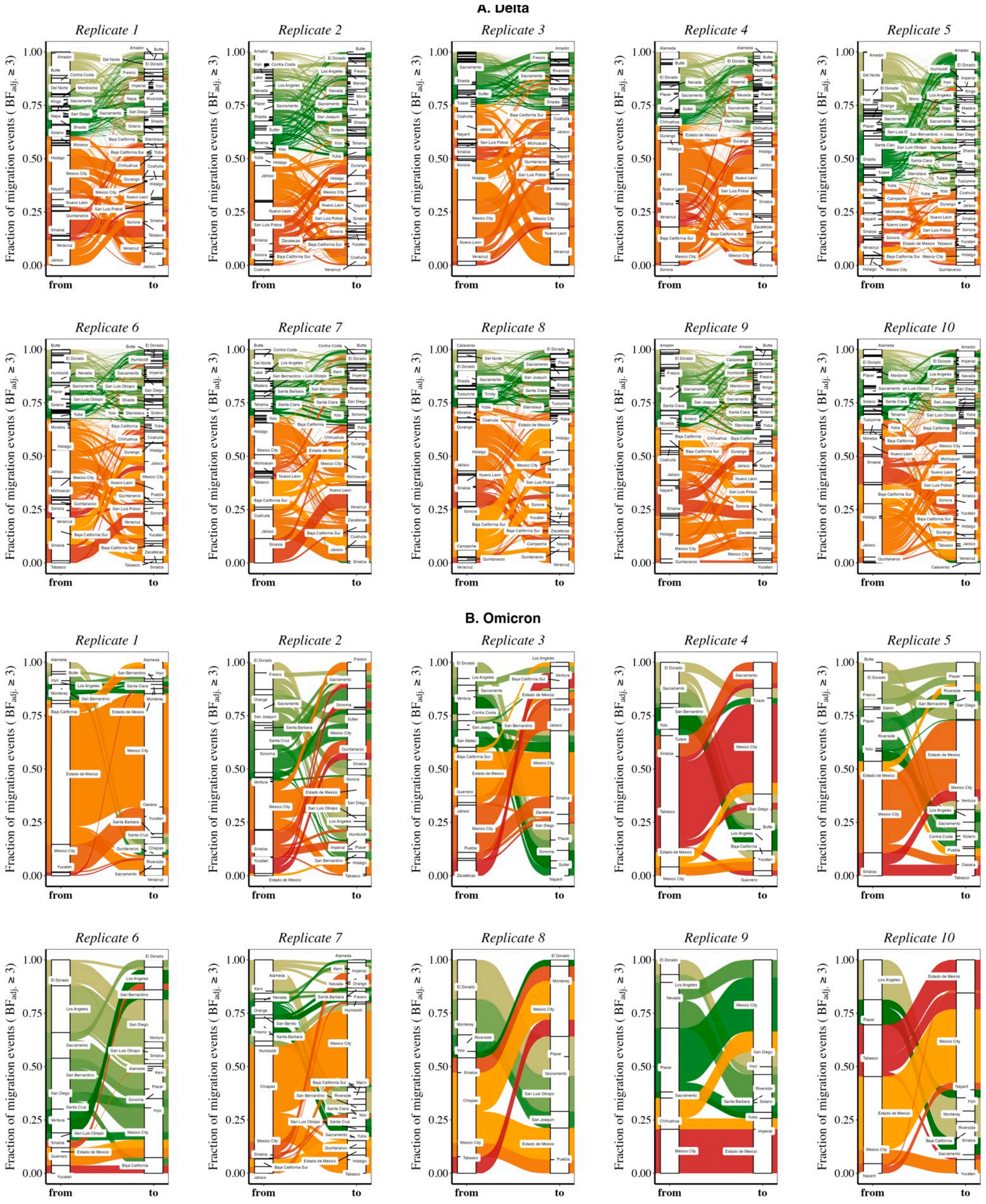

Supplementary Figure S8. Probability Density of variant migration events between locations for Delta (Panel A) and Omicron (Panel B) variants.

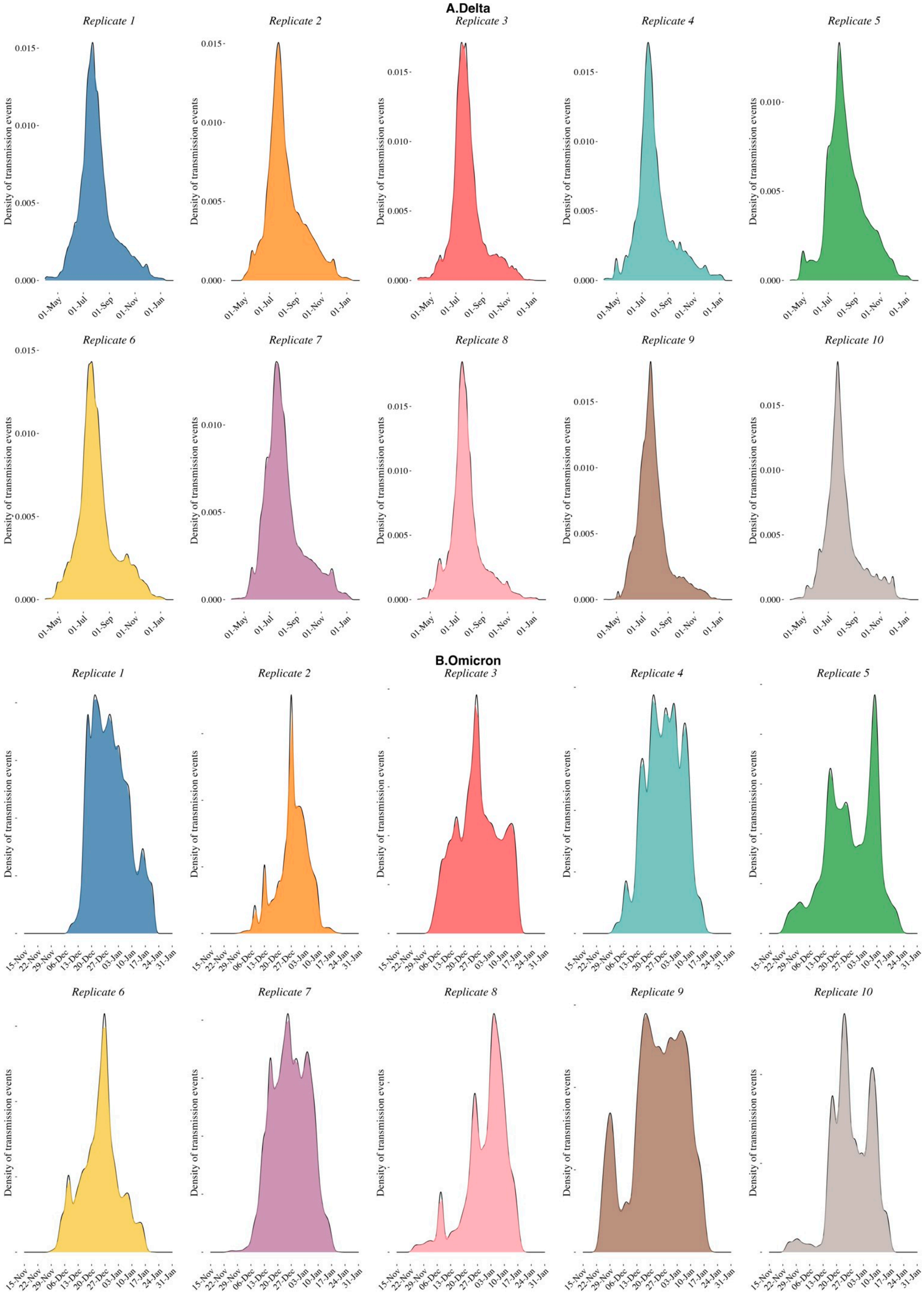

Supplementary Figure S9. Relative contribution of national vs international transmission events to the Delta (Panel A) and Omicron (Panel B) epidemics overtime.

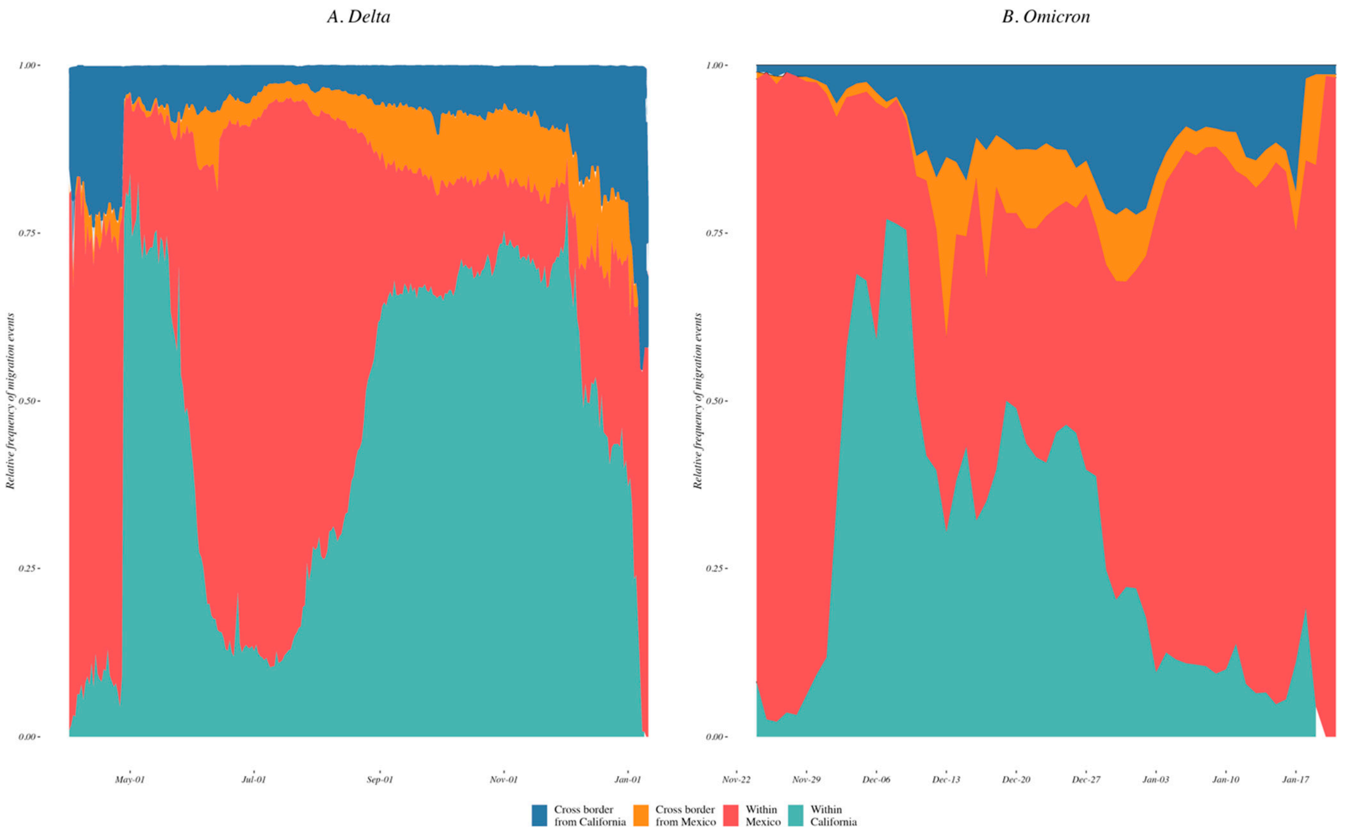

Supplementary Figure S10. Distribution of the geographic distances of phylogenetic lineage movement events for Delta (A) and Omicron (B) variants. The panel on the left combines the results of all 10 replicates shown on the right.

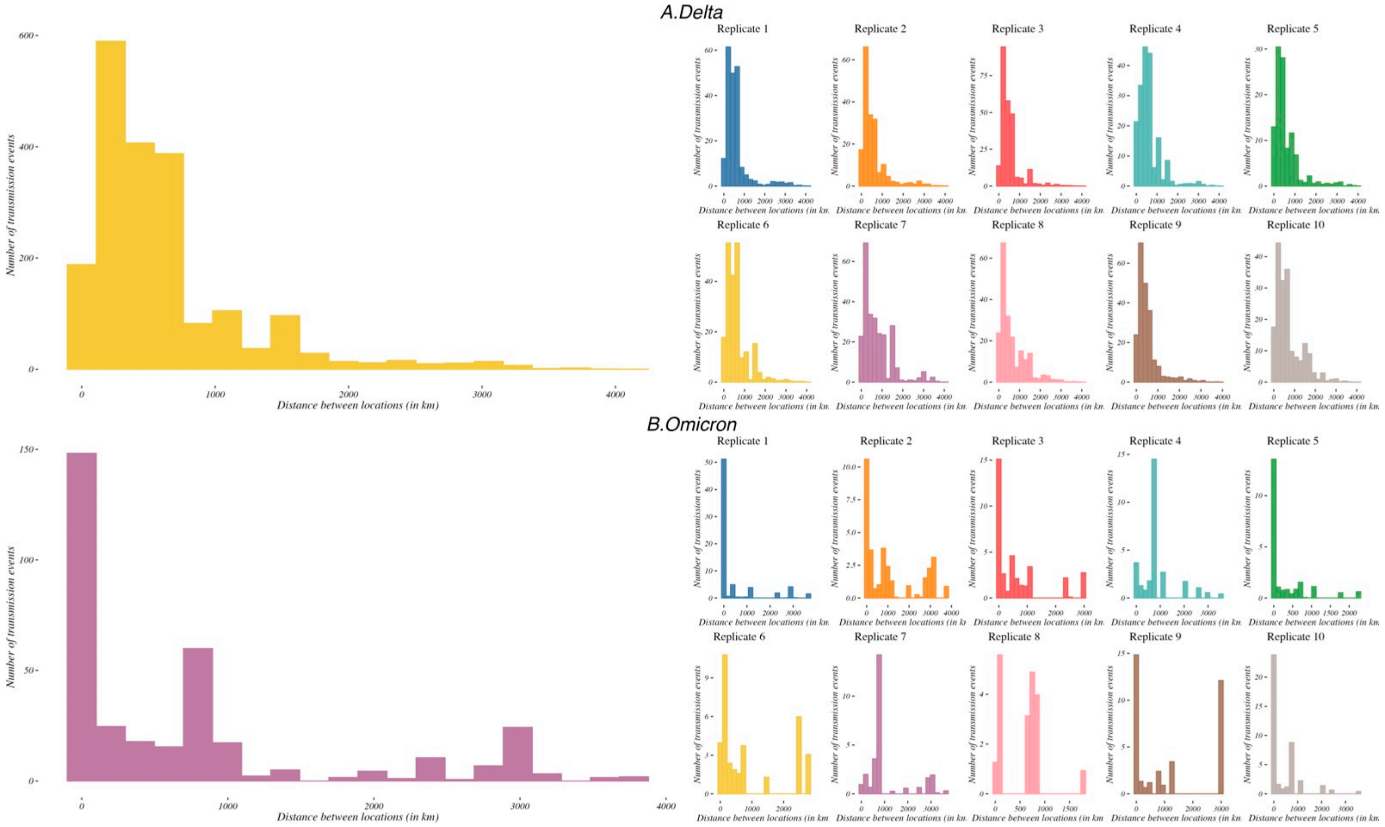

Supplementary Figure S11. Density of viral migration events across the US Mexico border and international travels.

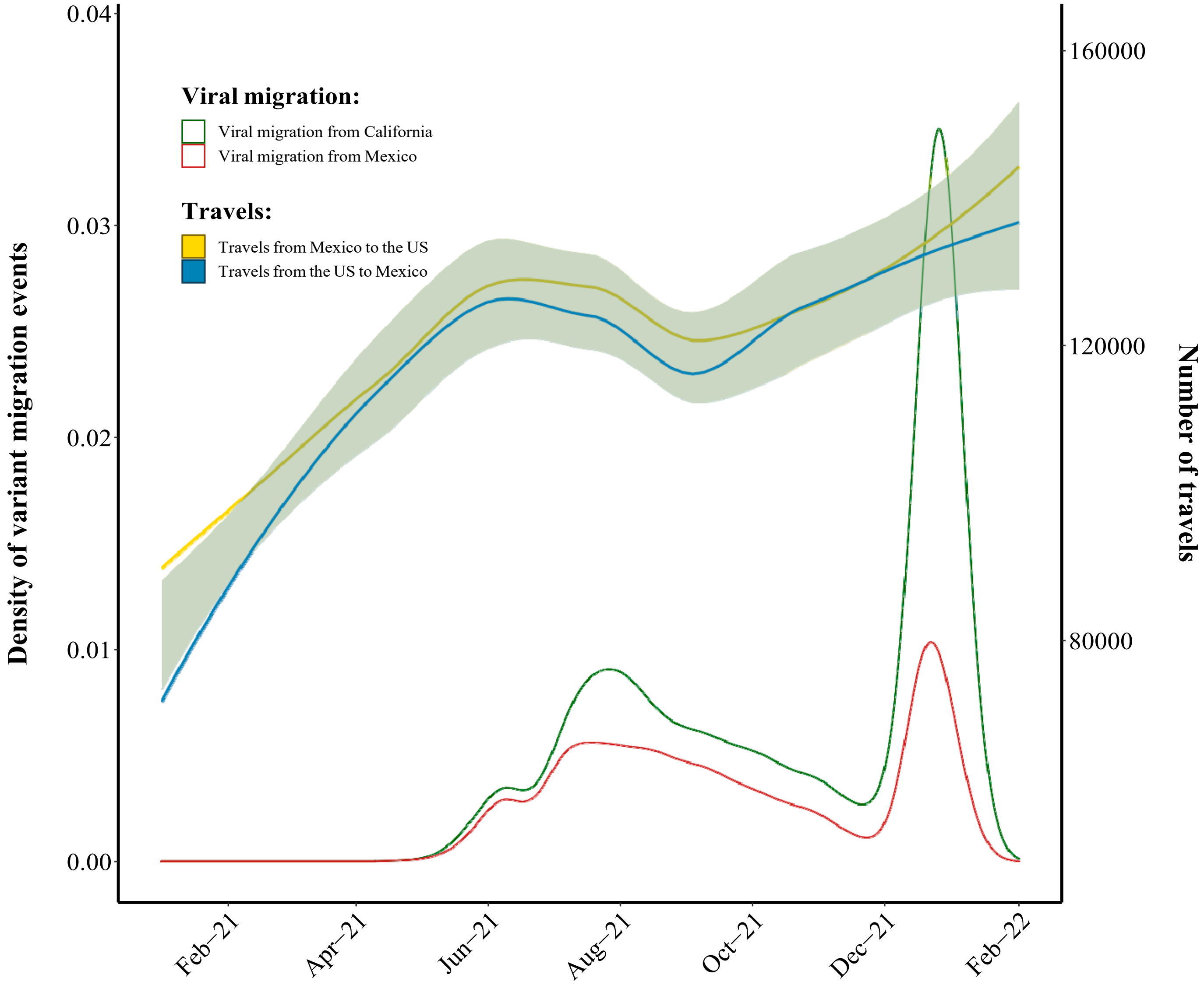

Supplement: Supplementary file 1 [file viruses-14-01494-s001.zip › viruses-1784784-supplementary.pdf]
